# Effects of Different Patterns and Sources of Trace Elements on Laying Performance, Tissue Mineral Deposition, and Fecal Excretion in Laying Hens

**DOI:** 10.3390/ani11041164

**Published:** 2021-04-19

**Authors:** Kaili Yang, Shengjun Hu, Rui Mu, Yiqing Qing, Liang Xie, Liyuan Zhou, Kolapo M. Ajuwon, Rejun Fang

**Affiliations:** 1 College of Animal Science and Technology, Hunan Agricultural University, No. 1 Nongda Road, Furong District, Changsha 410128, China; ykelly@stu.hunau.edu.cn (K.Y.); hsjtais@126.com (S.H.); murui27@163.com (R.M.); qingyq@stu.hunau.edu.cn (Y.Q.); axl853477258@163.com (L.X.); zly0430@126.com (L.Z.); 2 Hunan Co-Innovation Center of Animal Production Safety, Changsha 410128, China; 3 Department of Animal Sciences, Purdue University, West Lafayette, IN 47907-2041, USA; kajuwon@purdue.edu

**Keywords:** trace element, laying hens, performance, mineral deposition, fecal mineral excretion

## Abstract

**Simple Summary:**

This study aims to investigate the effects of laying performance, tissue mineral deposition, and fecal excretion from laying hens fed dietary treatments with different patterns and sources of trace elements including zinc (Zn), iron (Fe), copper (Cu), manganese (Mn), and selenium (Se). A total of 864 healthy laying hens with similar laying rate (Roman, 26-week-old) were randomly divided into nine treatments, with six replications of 16 birds per replication, including a control treatment (basic diet without added extra trace minerals) and four patterns with different element sources (inorganic mineral or organic mineral): 1. NRC (1994) recommended level pattern (NRC-L pattern); 2. NY/T 33-2004 recommended level pattern (NY/T-L pattern); 3. 50% NRC (1994) recommended level (50% NRC-L pattern); 4. the ratio of minerals in the blood of laying hens was taken as the supplement proportion of trace elements, and Zn was supplemented depended on the NRC recommended level pattern (TLB pattern). The results indicated that supplemental trace elements improved laying rate, liver, kidney, pancreas, spleen, pectorals muscle, and tibia mineral concentrations (Zn, Fe, Cu, Mn, Se). Patterns affected the tissue and fecal mineral concentrations. In addition, supplementation of the minerals with organic trace elements increased tissues and fecal mineral concentration than those with inorganic minerals. In conclusion, diet supplemented with the organic trace minerals of 50% NRC-L pattern for laying hens promoted optimum laying performance, mineral deposition, and reduced mineral excretion.

**Abstract:**

This study was conducted to investigate the effects of different patterns and sources of Zn, Fe, Cu, Mn, and Se on performance, mineral deposition (liver, kidney, pancreas, spleen, pectorals muscle, and tibia), and excretion of laying hens, then to find an optimal dietary supplemental pattern of trace elements in laying hens. A total of 864 healthy laying hens with similar laying rate (Roman, 26-week-old) were randomly divided into nine treatments, with six replications of 16 birds per replication, including a control treatment and four patterns with different element sources (inorganic or organic): (1) Control treatment (basic diet without added extra trace minerals, CT); pattern 1, NRC (1994) recommended level (NRC-L): (2) inorganic minerals of NRC-L pattern (IN), (3) organic minerals of NRC-L pattern (ON); pattern 2, NY/T 33-2004 recommended level (NY/T-L): (4) inorganic minerals of NY/T-L pattern (IY), (5) organic minerals of NY/T-L pattern (OY); pattern 3, 50% NRC (1994) recommended level (50% NRC-L): (6) inorganic minerals of 50% NRC-L pattern (IHN), (7) organic minerals of 50% NRC-L pattern (OHN); pattern 4, the ratio of minerals in blood of laying hens was taken as the supplement proportion of trace elements, and Zn was supplemented depended on NRC recommended level (TLB): (8) inorganic minerals of TLB pattern (IB), (9) organic minerals of TLB pattern (OB). Two weeks were allowed for adjustment to the conditions and then measurements were made over eight weeks. Supplementation of trace elements led to increased daily egg weight (*p* < 0.05). Patterns of minerals in diets affected the content of liver Mn, pancreas Mn, tibia Mn, and the tissues Se (*p* < 0.05). Sources of minerals had positive effects on daily egg weight (*p* < 0.05), the concentrations of liver Fe, kidney Cu, tissues Se (except spleen), and fecal Se (*p* < 0.05). In conclusion, diet supplemented with the organic trace minerals of 50% NRC-L pattern (OHN) in laying hens promoted optimum laying performance, mineral deposition, and reduced mineral excretion.

## 1. Introduction

Nowadays, the improvement of diet composition becomes a key factor to improve the health status and welfare of animals [1] as well as to enhance productivity in livestock [2,3,4]. Trace elements like zinc (Zn), iron (Fe), copper (Cu), manganese (Mn), and selenium (Se) play very essential roles in nutrition and physiology of animals [5]. As activators of various enzymes, these five trace elements participate in metabolism and transport substances in vivo, including increasing the membrane structure stability, antioxidant reaction, immune defense metabolism, the processes of reproduction, hormone synthesis, and bone growth [6,7,8,9].

For birds, trace minerals are important, while most feedstuffs do not contain adequate quantities of most trace elements. In order to fulfill the requirements of mineral elements for poultry, additional mineral additive elements must be added to the diet. There are authoritative recommendations like National Research Council (NRC, 1994) and Chinese Feeding Standard of Chicken (NY/T 33-2004). However, supplementation of trace elements has been overbalancing [10]. Excessive ingestion of trace elements not only affects the health of hens, but also increases the excretion of minerals into the soil or/and water, causing environmental contamination [11]. The utilization of minerals is a complex process which depends on the animals (breed and age), the bioavailability of mineral forms, the mineral interaction (synergism and antagonism), and so on [11,12].

Inorganic minerals are commonly used in livestock and poultry production, such as oxides or sulphates, due to low cost and availability [13]. However, during the passage through the digestive tract, ions from soluble inorganic trace elements can potentially be combined with and excreted with other dietary components, making them less available. [14]. Organic minerals contain metal ions chemically in connection with organics, which showed uniform stability and high bioavailability. Therefore, the trace elements seem to be more readily available in the gastrointestinal system due to forming complexes with other ration components [15]. Numerous studies have reported that organic trace elements improved laying performance, increased mineral absorption, increased the mineral concentrations of tissue, and decreased mineral excretion compared to inorganic trace elements. Different concentrations of supplemental trace elements also can affect the above-mentioned parameters [16,17,18].

Our group has proposed a concept of “Mineral Element Ideal Pattern (*MEIP*)”, the composition and proportion of mineral elements in the diet are consistent with the quality and quantity of mineral elements required by animals, with the expectation that an ideal pattern of dietary mineral elements will be optimally utilized by animals to support physiological functions [19]. Therefore, experiments are needed to determine optimum proportion of minerals in diets of animals to establish the ideal pattern. Determination of appropriate level and source of trace elements in diets of laying hens is a strategy for achieving optimal growth performance and reducing mineral excretion in feces. The mineral requirements of animals can be indicated by the concentration of minerals in tissues and blood [20]. Some organs are recognized as indicators for assessing the bioavailability of specific minerals. For example, bone zinc content can be considered as a responsive criterion for Zn bioavailability [21]. Liver and kidneys are sensitive to the variation of Cu concentration. Mineral metabolism, regulation, and storage can affect mineral concentration in spleen and liver [22]. It is still unknown whether supplementation of trace elements in the diets of laying hens at the same ratio in blood is beneficial or not. We hypothesized that lower supplementation of mineral elements than NRC recommendations will support normal production performance with no negative influence on the laying hens, and that organic trace minerals will have higher bioavailability. Therefore, this study was conducted to investigate the effects of different patterns and sources of trace elements on laying performance, tissue mineral element retention, and fecal mineral excretion of laying hens to determine the *MELP*.

## 2. Materials and Methods

### 2.1. Experimental Animals

This experiment was conducted on a commercial poultry farm (Yiyang, China). A total of 864 healthy laying hens (Roman pink, 26-week-old) with similar laying rate (95 ± 0.5%) body weight (1.9 ± 0.05 kg) were randomly divided into 9 treatments, with 6 pens of 16 birds per pens. This trial lasted for 10-wk, including 2 weeks for adaptation and 8 weeks for experiment. During the experiment, the birds were ad libitum to feed and water, with management of 16 L: 8D. Feeding management and vaccination programs were concordant to common practice.

### 2.2. Dietary Treatments

The basic diet was formulated suggested by NRC (1994) (Table 1). In this trial, there were basic diet and four patterns with two sources (inorganic or organic minerals) which were divided into nine treatments (Table 2): (1) Control treatment (basic diet without add extra trace minerals, **CT**); pattern 1, NRC (1994) recommended level (**NRC-L**): (2) inorganic trace minerals of NRC-L (**IN**) pattern, (3) organic trace minerals of NRC-L pattern (**ON**); pattern 2, NY/T 33-2004 recommended level (**NY/T-L**): (4) inorganic trace minerals of NY/T-L pattern (**IY**), (5) organic trace minerals of NY/T-L pattern (**OY**); pattern 3, 50% NRC (1994) recommended level (**50% NRC-L**): (6) inorganic trace minerals of 50% NRC-L pattern (**IHN**), (7) organic trace minerals of 50% NRC-L pattern (**OHN**); pattern 4, the ratio of mineral elements in blood of laying hens was taken as the supplement proportion of trace elements, and Zn was supplemented depended on NRC recommended level (**TLB**): (8) inorganic trace minerals of TLB pattern (**IB**) [23,24], (9) organic trace minerals of TLB pattern (**OB**). The inorganic Zn (>34.5%), Fe (>30%), Cu (>25.06%) and Mn (>31.8%) were in the form of feed-grade sulphate, and Se (>1%) was in form of sodium selenite. The organic Zn (>24%), Fe (>12%), Cu (>12%) and Mn (>14%) were provided as amino-acid minerals while the Se was as selenium yeast (Se > 0.2%), the organic trace elements (Zn, Fe, Cu, Mn, Se) were provided by Changsha Xingjia Biotechnology Share Co., Ltd. (Changsha, China).

### 2.3. Samples Collection and Measurement

#### 2.3.1. Laying Performance

Egg weight and number for each replicate were recorded daily. Feed consumption was recorded weekly after introduction of the experimental diets. At the end of the trial, laying rate, average egg weight (g/egg), average daily feed intake (ADFI, g/bird per day), and feed-to-egg ratio were calculated.

#### 2.3.2. Mineral Concentration

After 8 weeks on experimental diets, a bird was euthanized through cervical dislocation per replicate. Liver, kidney, pancreas, spleen, pectoral muscle, and tibias was collected and stored at −80 °C.

Tibia was immersed until fat was extracted, and then the traces of flesh and cartilages were removed. A quarter of feces were collected for 3 consecutive days at the end of the experiment for each replicate, dried at 65 °C for 48 h, and smashed and sifted to remove feathers. Then, the fecal sample was grounded to pass through a 1-mm screen after samples achieved constant weight and packed in self-locked plastic bags and stored at −20 °C for analysis.

The Zn, Fe, Cu, and Mn content of samples were analyzed by inductively coupled plasma optical emission spectrometry (ICP-OES, Optima 8300, PerkinElmer, Waltham, MA, USA) according to the method suggested by Qiu et al. [25]. The Se of samples were analyzed by atomic fluorescence meter (AFS-920, Titan, Beijing, China) according to the method suggested by Wang et al. [26].

### 2.4. Statistical Analysis

The data of the experiment were analyzed using one-way ANOVA by SPSS 23.0 (SPSS. Inc., Chicago, IL, USA) to compare all supplement experiment treatments with the control. Data were further analyzed as a 4 × 2 (pattern × source) factorial arrangement of treatments except control treatment by GLM using SPSS 23.0 (SPSS. Inc., Chicago, IL, USA), which included the main effects of pattern and source of trace elements, as well as their interaction. Dietary treatment was regarded as an independent variable. One dietary treatment was regarded as the experiment unit, with six replicates, and one replicate was regarded as a statistical unit. Differences were analyzed by Tukey’s multiple range tests whose significance levels were defined as *p* < 0.05, and a trend as 0.05 < *p <* 0.1. The data are expressed as means ± Standard Error of Mean (SEM).

## 3. Results

### 3.1. Laying Performance

The analyzed composition of trace minerals in diets is shown in Table 3. The concentrations of Zn, Fe, Cu, Mn, and Se in basic diets were 16.1, 302, 4.90, 17.9, and 0.06 mg/kg, respectively. As indicted in Table 4, compared with OB treatment, OHN and OB treatments increased the average egg weight (*p* < 0.05). No differences were detected on ADFI, laying rate, and feed-to-egg ratio (*p* > 0.10). The average egg weight was increased by organic minerals compared to inorganic form (*p* < 0.05). The average daily egg weight of hens fed dietary of OHN was 2.9% higher compared with the hens from CT group (*p* < 0.05). A supplemental trace element pattern did not affect laying performances (*p* > 0.10). There was a trend of interaction in average egg weight between pattern and source (0.05 < *p* <0.1).

### 3.2. Trace Elements Status on Tissues

The effects of trace elements at different patterns and sources on the liver minerals in Table 5. OHN treatment had higher liver Zn and Fe concentrations than CT and IY groups. OB treatment had the highest liver Se concentration among the treatments (*p* < 0.05). Birds fed organic trace elements diets increased liver Fe and Se content compared with those fed inorganic mineral diets (*p* < 0.05). Patterns of minerals affected the liver Mn and Se concentrations (*p* < 0.05). The NY/T-L pattern increased the accumulation of liver Mn compared with the TLB pattern (*p* < 0.05). The highest liver Se concentration was found in the TLB pattern (*p* < 0.05). In kidneys (Table 6), compared with CT treatment, the kidney Cu content was improved by ON treatment (*p* < 0.05). Kidney Se content was the highest in the OB treatment (*p* < 0.05). In addition, kidney Se concentration was significantly higher in hens fed diets of OY and IB treatments than other treatments (*p* < 0.05). Supplementation of trace elements as organic form increased kidney Cu and Se concentrations compared to those fed inorganic mineral treatments (*p* < 0.05). Patterns of minerals significantly influenced kidney Se concentration (*p* < 0.001), while it had tendencies for affecting both Zn and Mn concentrations (0.05 < *p* <0.1). Kidney Se content in hens fed diet of TLB pattern was highest. In the pancreas (Table 7), Se concentration in OB groups was the highest among these groups (*p* < 0.05). The pancreas of birds in OHN treatment had higher Se content than that in other treatments except the OB group (*p* < 0.05). Hens of OY and ON treatments showed higher pancreas Mn content than OB treatment (*p* < 0.05). The pattern of minerals had influences on the pancreas Mn and Se concentrations (*p* < 0.05). Among mineral patterns, pancreas Mn content was significantly reduced while Se concentration was increased in birds fed a diet supplemented with a trace element of the TLB pattern (*p* < 0.05). It had a tendency for a 50% NRC-L pattern to increase pancreas Zn content (0.05 < *p* <0.1). The Se concentration from IB and OB treatments were higher than that from other treatments in spleen (*p* < 0.001). Spleen Se content in OY and OHN treatments were higher than that from CT, IN, ON, IY, and IHN treatments (*p* < 0.001) (Table 8). Compared with the CT group, pectorals muscle Se concentration was increased in ON, OY, OHN, IB, and OB groups (*p* < 0.001) (Table 9). The spleen and pectorals muscle Se content was increased by adding supplemental organic trace elements of TLB patterns (*p* < 0.001). There was a trend for spleen Fe concentration in patterns and sources of trace elements (0.05 < *p* <0.1). The effects of trace elements at different patterns and sources on the tibia minerals in Table 10. The Tibia Mn concentration was improved by OY treatment than that in OB treatment (*p* < 0.05). Tibia Se concentration was the highest in supplemental trace elements of OB treatments (*p* < 0.001). Compared with CT, IN, ON, IY, IHN, and OHN treatments, birds fed OY and IB diets increased tibia Se content (*p* < 0.05). Supplementation of the diets with organic trace elements significantly increased tibia Se concentration than those with inorganic minerals (*p* < 0.001). Patterns of minerals influenced tibia Mn and Se concentrations (*p* < 0.05). The highest Mn and Se content in tibia were found in birds fed diets supplemented with trace minerals of NY/T-L pattern and TLB pattern, respectively (*p* < 0.05). The interaction between trace element patterns and sources was significant in light of liver Se, kidney Se, pancreas Se and Mn, pectorals muscle Se, and tibia Se (*p* < 0.05).

### 3.3. Fecal Mineral Concentration

The effects of different patterns and sources on fecal mineral excretion are presented in Table 11. Supplementation of the basal diet with trace minerals increased fecal Zn concentration (*p* < 0.05). IY and OY treatments significantly enhanced the Zn content compared to other groups (*p* < 0.05). The fecal Fe content in hens fed diets supplemented with trace minerals of IB treatment was higher than that in CT, IN, ON, IY, IHN, and OHN treatments (*p* < 0.05). Supplementation of the basic diet with trace minerals of ON, IY, OY, IB, and OB treatments significantly increased fecal Cu content compared with CT treatment (*p* < 0.05). Fecal Cu content in IY and OY treatments significantly increased compared with other treatments (*p* < 0.05), since the fecal Mn content was found to be significantly higher in hens fed diets of IN, ON, IY, OY, and IHN treatments than CT treatment (*p* < 0.05). Compared with CT treatment, fecal Se concentration was increased with IY, OY, IB, and OB treatments (*p* < 0.05) while decreased with IN, ON, IHN, and OHN treatments (*p* < 0.05). The patterns of minerals influenced fecal Zn, Fe, Cu, Mn, and Se content (*p* < 0.001). Among these patterns, the mineral concentration of feces was lower in patterns of 50% NRC-L and NRC-L treatments. The highest fecal Zn and Mn concentrations were detected in supplementation of trace minerals of NY/T-L patterns (*p* < 0.05), while Fe and Se concentrations were highest in TLB pattern groups (*p* < 0.05). Cu content in the excreta of hens were significantly increased (*p* < 0.001) with supplementation of trace mineral of NY/T-L and TLB pattern compared with the hens fed NRC-L and 50% NRC-L pattern (*p* < 0.001). Mn concentration was significantly decreased with TLB pattern treatment (*p* < 0.001). Mineral concentration in NRC-L pattern didn’t differ from those in 50% NRC-L patterns (*p* > 0.10). Compared with inorganic source, supplementation of organic trace element increased Cu and Se concentration of feces (*p* < 0.05). However, the lowest Cu and Se concentration in feces was found in birds fed a diet of an organic 50% NRC-L pattern (OHN), and the highest fecal Se content was found in organic TLB pattern treatment. The interaction between the patterns and sources was significant in terms of fecal Zn, Fe, and Se concentration (*p* < 0.05).

## 4. Discussion

Compared with Zn, Fe, Cu, Mn, and Se supplemental recommended content of NRC (1994) to laying hens, mineral concentration of basial diet in current study exhibited that Zn and Mn concentrations were lower, Fe and Cu content was higher, and Se concentration was approximate. Zn content was lower while Fe, Cu, Mn, and Se content was higher in basic diets than in supplemental recommendation of trace minerals with the 50% NRC pattern. Compared with the supplemental trace minerals of recommendation of NY/T 33-2004, Zn, Cu, Mn, and Se concentrations were deficient while Fe was rich in basial diets. Zn, Cu, and Se concentrations were lower while Fe and Mn concentrations were higher in basial diets compared with recommended trace elements amount of the TLB pattern.

The performance of laying hens is mediated by several factors, including the metabolism of protein, carbohydrate and energy in cells, and tissues and organs which are directly or indirectly involved in Zn, Fe, Cu, Mn, and Se [27]. The different adding amount and forms of trace elements have different influences on the growth performance [28]. Dikmen [29] showed that the addition of amino acid compound Zn and Mn in the diets of laying hens could significantly improve the egg weight and egg yield. Liu et al. [30] observed Se levels (0.3 and 0.5 mg/kg) and sources (Yeast selenium and sodium selenite) had no difference in egg weight and feed conversion, but ADFI was improved by 0.3 mg/kg Se of yeast selenium, and an interaction of Se sources and levels was in ADFI. Our results exhibited that different sources of trace elements (Zn, Fe, Cu, Mn, and Se) had a significant influence on average egg weight of laying hens, and the organic forms of trace elements showed a higher laying performance. In the present study, OHN treatment had the highest average egg weight, but had no influence on feed intake, laying rate, egg ratio, and our results are in accordance with Gheisari et al. [31]. In contrast, in the study of Gheisari et al. [31], feed intake and feed conversion ratio were significantly affected by forms and dosage of Mn, Zn, and Cu. Ramos-Vidales et al. [17] reported that the treatments of organic trace elements (Carbo-Amino-Phosphate-Chelates) had no significant effect on the ratio of feed to egg, egg yield, and egg weight production performance of laying hens. The reasons for the difference may attributed to composition of basal diet, the concentration and sources of trace elements, interaction between minerals, and daily management of layers [32,33].

Trace elements can deposit into the liver, kidney, pancreas, bone, and other internal organs of the animal body. The physiological changes of the body over a certain period and the biological efficacy of dietary trace elements usually reflected the content of trace elements in tissues and organs. [11,34]. As Abedini et al. [12] investigated, Zn content in the tibia, liver, pancreas, and eggs of laying hens was significantly increased with the organic Zn (Zn-methionine) and nano Zn treatments, compared with the control group (basic diet without mineral supplementation). Wang et al. [26] showed that the addition of 62.5% commercial recommended amounts (Cu, Zn, Fe, Mn, and Se, 10, 80, 30, 80, and 0.3 mg/kg, respectively) of organic trace elements (metal proteinates were sequestered with enzymatically hydrolyzed soybean protein) promoted the deposition of trace elements in liver, pancreas, and other tissues. Similar to the previous study, our findings indicated that the organic mineral treatments could increase the trace elements content in tissues. This is because inorganic trace elements have poor bioavailability compared with the organic form. The high bioavailability of mineral chelates prevents chemical actions in the gastrointestinal tract and retains stability depending on their structure. Compared with organic mineral, chelates of trace elements pass the intestinal wall more effectively because of negative charge and amino acid transport of intestinal wall [11,15]. The different trace element patterns and sources had significant effects on the trace elements content in diverse tissues. Trace mineral homeostasis is maintained in tissues which may serve as biomarkers of intake and status [34]. In this study, different levels of Cu, Zn, Fe, Mn, and Se were found in various tissues. It also observed Zn and Fe content in liver, Cu content in kidney, Mn content in pancreas and tibia, and Se in tissues had significantly changed under the influence of different patterns and sources. The Se and Cu content in diets of NRC-L (IN and ON) and 50% NRC-L (IHN and OHN) patterns, Zn and Fe concentrations in NY/T-L (IY and OY) diets of pattern, and Fe, Cu, and Se concentrations in the diets of TLB patterns (IB and OB) were higher, while dietary Mn content in the diets of the TLB pattern was lower. The Zn, Fe, Cu, Mn, and Se concentrations in the basic diet were the lowest. Corresponding to the supplemental dietary mineral concentration, the TLB pattern increased tissues Se concentration, while it decreased the Mn content relatively. However, the highest tissues Cu concentration were found in NY/T-L pattern treatment. In general, the present study showed that the OHN treatment increased the mineral content in tissues. Similarly, Qiu et al. [25] mentioned that low doses of trace elements in organic forms (proteinated trace minerals) improved minerals (Fe, Mn, Zn) deposition. There are interactions among elements. The previous results revealed that Zn is the first limiting mineral among Cu, Fe, Mn, and Zn [35]. Cu and Fe both have antagonistic and synergistic effects [36]. An excess of Cu will hinder the absorption of Fe and Mn in the body. Se and Mn in the body are negatively correlated [37,38]. This might be the reason that the diets of TLB pattern with the high Fe and Cu content had no significant influence on the Fe and Cu deposition in the body. The 50% NRC pattern had the same ratio as the NRC pattern, which exhibited different performances due to antagonistic and synergistic of minerals possibly. Moreover, it’s better to reduce the Cu and Se content of TLB diet, while increasing the Mn content of the TLB diet.

In this study, the Zn, Fe, Cu, Mn, and Se content in the feces of laying hens were significantly affected by different patterns of trace elements (Zn, Fe, Cu, Mn, and Se). It was observed that fecal trace elements were reduced with a lower dosage of trace minerals in the feedstuff. Zn, Fe, Cu, and Se content of hen excreta were lower in the treatments of NRC-L and 50% NRC-L pattern than other patterns. Fecal Cu concentration was reduced by the TLB pattern. Previous investigations suggested that a positive correlation exists between the excretion and feedstuff of trace element content [26,39]. Similarly, the research of Zhu et al. [40] found that Cu, Zn, Fe, and Mn content in chicken feces were significantly reduced by low dose trace elements (Cu, Zn, Fe, and Mn are 3, 24, 12, and 20 mg/kg, respectively). However, there was no significant difference between organic (amino plex) and inorganic (sulphates) forms. In the current study, Zn, Fe, and Mn concentrations were not affected by mineral sources while Cu and Se did. Compared with organic mineral, fecal Cu content was reduced while Se concentration was increased with inorganic form. However, on the contrary, supplemental organic trace elements of 50% NRC-L pattern (OHN) reduced Cu content in the excreta of hens, while organic trace elements of TLB (OB) pattern increased fecal Se content. Among these patterns, Cu concentration was lower in the diets of the 50% NRC-L pattern. On the other hand, Se concentration was higher in diets with TLB patterns. It showed a lower dosage of supplemental organic minerals in the scope of supplemented concentration of trace elements for diets of hens, which may promote absorption of trace elements while reducing fecal mineral content. If mineral supplementation is out of range, fecal trace elements will be increased in hens fed diets supplemented with organic mineral than those fed inorganic minerals. Organic trace elements are supplemented at lower levels in diets of animals because they have higher bioavailability compared with inorganic forms [34,41]. Yenice et al. [15] showed that the Cu, Mn, and Zn content in the feces of laying hens were significantly reduced by organic Cu, Mn, and Zn (methionine chelated mineral) in lower levels (2.5, 40, and 30 mg/kg) compared with inorganic forms (Cu, sulfate source; Zn, Mn, oxide sources). This research revealed that the OHN diet significantly reduced Zn, Cu, and Se concentrations of excretion, and the IHN treatment had the lowest fecal Fe content. As reported in other studies, the organic minerals reduced fecal Fe, Cu, Mn, and Zn concentrations of laying hens because of the high bioavailability compared with inorganic trace elements [15,25,42]. This study showed that the use of lower levels of organic trace minerals may lead to lower excretion of minerals in feces, helping to reduce the negative impact of poultry production on the environment.

## 5. Conclusions

This study aimed to provide a basic guide for the effective utilization of minerals on poultry production. We found that the diets of supplemental trace element of NY/T-L pattern increased tissue Mn content while trace elements of TLB pattern increased Se concentration. Organic trace minerals increased laying performance, mineral deposition in tissues, and reduction of fecal mineral excretion. In addition, OHN improved egg weight, OY and OHN improved mineral deposition in tissues, and OHN reduced fecal mineral excretion in laying hens. In conclusion, the organic trace minerals of 50% NRC-L (OHN) in laying hen diets promoted optimal laying performance, mineral deposition, and reduced mineral excretion. In poultry production, adding low dosage minerals with organic forms can improve bird performance and mineral bioavailability within limits, but it should pay more attention to the interactions among the trace elements.

## Figures and Tables

**Table 1:** Composition and nutrition of basal diet (as-fed basis).

Ingredient	Content (%)	Nutrient Level	Content
Corn	60.50	Calculated compositions	
Soybean meal	24.00	ME, MJ/kg ^2^	11.08
Soybean oil	1.00	CP, % ^3^	16.00
Rapeseed meal	2.40	Lysine, %	0.81
Limestone	8.80	Methionine, %	0.36
CaHPO_4_	1.30	Calcium, %	3.60
Premix ^1^	2.00	Total phosphorus, %	0.55
Total	100.00	Analyzed compositions	
		CP, %	16.43
		Calcium, %	3.40
		Total phosphorus, %	0.54

^1^ Premix feed provided per kilogram of diet: Vitamin A 6000 IU, Vitamin D3 2775 IU, Vitamin E 25.0 mg, Vitamin K3 2.25 mg, Vitamin B1 1.8 mg, Vitamin B2 4.50 mg, Vitamin B6 4.0 mg, Vitamin B12 0.20 mg, pantothenic acid 12.0 mg, niacin acid 25 mg, biotin 0.14 mg, folic acid 0.8 mg, phytase 1000 IU. ^2^ ME: metabolic energy. ^3^ CP: crude protein.

**Table 2:** Trace element addition pattern ^1^.

Pattern	Treatments	Zn	Fe	Cu	Mn	Se
	CT	0	0	0	0	0
NRC-L	IN	100.00	174.43	11.42	85.71	0.29
		35.00	45.00	4.00	20.00	0.05
	ON	100.00	174.43	11.42	85.71	0.29
		35.00	45.00	4.00	20.00	0.05
NY/T-L	IY	100.00	75.00	10.00	75.00	0.38
		80.00	60.00	8.00	60.00	0.30
	OY	100.00	75.00	10.00	75.00	0.38
		80.00	60.00	8.00	60.00	0.30
50% NRC-L	IHN	100.00	174.43	11.42	85.71	0.29
		17.50	22.50	2.00	10.00	0.025
	OHN	100.00	174.43	11.42	85.71	0.29
		17.50	22.50	2.00	10.00	0.025
TLB	IB	100.00	358.21	38.20	21.21	2.57
		35.00	125.37	13.37	7.42	0.90
	OB	100.00	358.21	38.20	21.21	2.57
		35.00	125.37	13.37	7.42	0.90

^1^ In each pattern, upper data mean ratio of one trace mineral element to zinc (as 100.00) and down data mean of the supplemental level of this trace mineral element in diets (mg/kg). Control treatment (basic diet without add extra trace minerals, **CT**); NRC recommended level (**NRC-L**), inorganic trace minerals of NRC-L (**IN**), organic trace minerals of NRC-L pattern (**ON**), NY/T 33-2004 recommended level (**NY/T-L**), inorganic trace minerals of NY/T-L pattern (**IY**), organic trace minerals of NY/T-L pattern (**OY**); pattern 3, 50% NRC (1994) recommended level (**50% NRC-L**), inorganic trace minerals of 50% NRC-L pattern (**IHN**), organic trace minerals of 50% NRC-L pattern (**OHN**), the ratio of mineral elements in blood of laying hens was taken as the supplement proportion of trace elements, and Zn was supplemented depended on NRC recommended level (**TLB**), inorganic trace minerals of TLB pattern (**IB**), organic trace minerals of TLB pattern (**OB**).

**Table 3:** Trace elements analyzed content of diets (mg/kg, in trace elements).

Patterns		NRC-L		NY/T-L		50% NRC-L		TLB	
Items	CT	IN	ON	IY	OY	IHN	OHN	IB	OB
Zn	16.1	45.8	46.3	92.1	94.6	35.5	34.6	46.8	46.2
Fe	302	353	348	379	384	331	328	409	406
Cu	4.90	8.55	8.93	10.7	11.8	7.44	7.41	12.4	12.9
Mn	17.9	34.2	34.4	70.1	73.8	27.2	25.9	23.0	24.4
Se	0.06	0.10	0.13	0.35	0.36	0.08	0.08	1.14	1.20

Control treatment (basic diet without add extra trace minerals, **CT**); NRC recommended level (**NRC-L**), inorganic trace minerals of NRC-L (**IN**), organic trace minerals of NRC-L pattern (**ON**), NY/T 33-2004 recommended level (**NY/T-L**), inorganic trace minerals of NY/T-L pattern (**IY**), organic trace minerals of NY/T-L pattern (**OY**); pattern 3, 50% NRC (1994) recommended level (**50% NRC-L**), inorganic trace minerals of 50% NRC-L pattern (**IHN**), organic trace minerals of 50% NRC-L pattern (**OHN**), the ratio of mineral elements in blood of laying hens was taken as the supplement proportion of trace elements, and Zn was supplemented depended on NRC recommended level (**TLB**), inorganic trace minerals of TLB pattern (**IB**), organic trace minerals of TLB pattern (**OB**).

**Table 4:** Effect of different patterns and sources of mineral elements on laying performance ^1^.

Items		ADFI (g/Bird/per Day)	Laying Rate (%)	Average Egg Weight (g/Egg)	Feed-To-Egg Ratio
	CT	107.85	96.18	57.69 ^ab^	1.95
NRC-L	IN	107.53	95.52	57.39 ^ab^	1.98
	ON	106.60	95.97	57.17 ^ab^	1.95
NY/T-L	IY	106.82	94.55	57.36 ^ab^	1.97
	OY	106.75	94.61	57.65 ^ab^	1.99
50% NRC-L	IHN	106.49	95.50	57.63 ^ab^	1.94
	OHN	107.58	95.73	59.34 ^a^	1.93
TLB	IB	106.20	95.38	56.34 ^b^	1.98
	OB	110.58	96.39	58.69 ^a^	1.95
	SEM	0.38	0.32	0.20	0.07
Patterns	NRC-L	107.06	95.74	57.28	1.97
	NY/T-L	106.79	94.58	57.50	1.98
	50% NRC-L	107.04	95.61	58.48	1.93
	TLB	108.39	95.88	57.51	1.97
Sources	Inorganic	106.76	95.24	57.18 ^†^	1.97
	Organic	107.88	95.67	58.21	1.96
*p*-value	CT-trace element supplemented	0.164	0.929	0.008	0.808
	Pattern	0.458	0.579	0.113	0.347
	Source	0.155	0.541	0.009	0.548
	Pattern × Source	0.094	0.968	0.069	0.841

^1^ Data are given as treatment means; *n* = 6 pens per treatment, † different from the corresponding inorganic treatments (*p* < 0.05). Control treatment (basic diet without add extra trace minerals, **CT**); NRC recommended level (**NRC-L**), inorganic trace minerals of NRC-L (**IN**), organic trace minerals of NRC-L pattern (**ON**), NY/T 33-2004 recommended level (**NY/T-L**), inorganic trace minerals of NY/T-L pattern (**IY**), organic trace minerals of NY/T-L pattern (**OY**); pattern 3, 50% NRC (1994) recommended level (**50% NRC-L**), inorganic trace minerals of 50% NRC-L pattern (**IHN**), organic trace minerals of 50% NRC-L pattern (**OHN**), the ratio of mineral elements in blood of laying hens was taken as the supplement proportion of trace elements, and Zn was supplemented depended on NRC recommended level (**TLB**), inorganic trace minerals of TLB pattern (**IB**), organic trace minerals of TLB pattern (**OB**). Means for treatments within a row not showing a common small letter superscript differ (*p* < 0.05), † different from the corresponding inorganic treatments (*p* < 0.05).

**Table 5:** Effect of different patterns and Sources of mineral elements on liver (mg/kg) ^1^.

Items		Zn	Fe	Cu	Mn	Se
	CT	39.44 ^b^	67.34 ^b^	4.75	2.23	0.41 ^b^
NRC-L	IN	49.47 ^ab^	73.81 ^ab^	5.04	2.49	0.34 ^b^
	ON	53.52 ^ab^	81.32 ^ab^	5.69	2.69	0.31 ^b^
NY/T-L	IY	43.33 ^b^	68.37 ^b^	5.33	2.47	0.39 ^b^
	OY	52.29 ^ab^	86.47 ^ab^	5.55	2.75	0.45 ^b^
50% NRC-L	IHN	50.58 ^ab^	70.11 ^ab^	5.21	2.28	0.31 ^b^
	OHN	63.93 ^a^	90.23 ^a^	5.47	2.52	0.42 ^b^
TLB	IB	50.89 ^ab^	81.78 ^ab^	5.41	2.07	0.42 ^b^
	OB	47.02 ^ab^	83.90 ^ab^	5.22	1.98	0.82 ^a^
	SEM	1.64	1.85	0.08	0.06	0.03
Patterns	NRC-L	51.49	77.56	5.36	2.59 ^A^	0.32 ^B^
	NY/T-L	47.81	77.42	5.47	2.61 ^A^	0.41 ^B^
	50% NRC-L	57.26	80.17	5.34	2.40 ^AB^	0.37 ^B^
	TLB	49.15	82.84	5.31	2.02 ^B^	0.62 ^A^
Sources	Inorganic	48.67	73.5 ^†^	5.26	2.33	0.36 ^†^
	Organic	54.19	85.5	5.48	2.49	0.50
*p*-value	CT-trace element supplemented	0.017	0.004	0.192	0.071	<0.001
	Pattern	0.180	0.667	0.940	0.013	<0.001
	Source	0.108	0.001	0.225	0.238	<0.001
	Pattern × Source	0.299	0.209	0.460	0.755	0.001

^1^ Data are given as treatment means; *n* = 6 pens per treatment. ^a,b^ Means for treatments within a row not showing a common small letter superscript differ (*p* < 0.05), means for patterns within a row not showing a common capital letter superscript differ (*p* < 0.05), † different from the corresponding inorganic treatments (*p* < 0.05), the same as below. Control treatment (basic diet without add extra trace minerals, **CT**); NRC recommended level (**NRC-L**), inorganic trace minerals of NRC-L (**IN**), organic trace minerals of NRC-L pattern (**ON**), NY/T 33-2004 recommended level (**NY/T-L**), inorganic trace minerals of NY/T-L pattern (**IY**), organic trace minerals of NY/T-L pattern (**OY**); pattern 3, 50% NRC (1994) recommended level (**50% NRC-L**), inorganic trace minerals of 50% NRC-L pattern (**IHN**), organic trace minerals of 50% NRC-L pattern (**OHN**), the ratio of mineral elements in blood of laying hens was taken as the supplement proportion of trace elements, and Zn was supplemented depended on NRC recommended level (**TLB**), inorganic trace minerals of TLB pattern (**IB**), organic trace minerals of TLB pattern (**OB**).

**Table 6:** Effect of different patterns and Sources of mineral elements on kidneys (mg/kg).

Items		Zn	Fe	Cu	Mn	Se
	CT	29.33	62.34	3.60 ^b^	2.86	0.27 ^d^
NRC-L	IN	28.32	51.64	3.73 ^ab^	2.40	0.26 ^d^
	ON	31.79	58.79	4.17 ^a^	2.60	0.37 ^cd^
NY/T-L	IY	27.29	63.34	3.89 ^ab^	2.54	0.37 ^cd^
	OY	26.18	53.84	3.87 ^ab^	2.70	0.51 ^b^
50% NRC-L	IHN	25.73	50.63	3.95 ^ab^	2.38	0.29 ^d^
	OHN	27.37	60.42	4.09 ^ab^	2.18	0.41 ^c^
TLB	IB	28.69	53.8	3.97 ^ab^	2.10	0.57 ^b^
	OB	29.06	70.03	4.12 ^ab^	1.97	1.05 ^a^
	SEM	0.52	1.86	0.47	0.08	0.05
Patterns	NRC-L	30.06	55.22	3.95	2.50	0.31 ^C^
	NY/T-L	26.73	58.59	3.88	2.62	0.44 ^B^
	50% NRC-L	26.55	55.52	4.02	2.28	0.35 ^C^
	TLB	28.88	61.91	4.04	2.03	0.81 ^A^
Sources	Inorganic	27.51	54.85	3.89 ^†^	2.36	0.37 ^†^
	Organic	28.60	60.77	4.06	2.36	0.58
*p*-value	CT-trace element supplemented	0.167	0.220	0.042	0.089	<0.001
	Pattern	0.070	0.573	0.500	0.058	<0.001
	Source	0.294	0.137	0.044	0.971	<0.001
	Pattern × Source	0.449	0.139	0.287	0.722	<0.001

^a,b,c,d,A,B,C^ Means for treatments within a row not showing a common small letter superscript differ (*p* < 0.05), means for patterns within a row not showing a common capital letter superscript differ (*p* < 0.05), † different from the corresponding inorganic treatments (*p* < 0.05), the same as below. Control treatment (basic diet without add extra trace minerals, **CT**); NRC recommended level (**NRC-L**), inorganic trace minerals of NRC-L (**IN**), organic trace minerals of NRC-L pattern (**ON**), NY/T 33-2004 recommended level (**NY/T-L**), inorganic trace minerals of NY/T-L pattern (**IY**), organic trace minerals of NY/T-L pattern (**OY**); pattern 3, 50% NRC (1994) recommended level (**50% NRC-L**), inorganic trace minerals of 50% NRC-L pattern (**IHN**), organic trace minerals of 50% NRC-L pattern (**OHN**), the ratio of mineral elements in blood of laying hens was taken as the supplement proportion of trace elements, and Zn was supplemented depended on NRC recommended level (**TLB**), inorganic trace minerals of TLB pattern (**IB**), organic trace minerals of TLB pattern (**OB**).

**Table 7:** Effect of different patterns and sources of mineral elements on the pancreas (mg/kg).

Items		Zn	Fe	Cu	Mn	Se
	CT	33.13	19.12	1.79	1.96 ^ab^	0.20 ^e^
NRC-L	IN	38.04	24.39	1.84	2.21 ^ab^	0.19 ^e^
	ON	39.48	23.73	1.87	2.56 ^a^	0.28 ^d^
NY/T-L	IY	31.55	41.98	1.73	2.24 ^ab^	0.22 ^e^
	OY	38.71	21.12	1.89	2.75 ^a^	0.38 ^c^
50% NRC-L	IHN	44.40	19.45	1.86	2.30 ^ab^	0.21 ^e^
	OHN	46.93	21.44	2.1	2.00 ^ab^	0.50 ^b^
TLB	IB	40.05	20.85	1.91	1.96 ^ab^	0.30 ^d^
	OB	30.63	24.56	1.88	1.52 ^b^	0.97 ^a^
	SEM	1.49	2.42	0.04	0.09	0.01
Patterns	NRC-L	38.76	24.06	1.86	2.39 ^A^	0.24 ^D^
	NY/T-L	35.13	31.55	1.81	2.49 ^A^	0.30 ^C^
	50% NRC-L	45.66	20.45	1.98	2.49 ^AB^	0.37 ^B^
	TLB	35.34	22.70	1.89	1.74 ^B^	0.64 ^A^
Sources	Inorganic	38.51	26.67	1.83	2.18	0.23 ^†^
	Organic	38.94	22.71	1.93	2.20	0.54
*p*-value	CT-trace element supplemented	0.097	0.536	0.681	0.015	<0.001
	Pattern	0.051	0.528	0.604	0.002	<0.001
	Source	0.878	0.481	0.280	0.813	<0.001
	Pattern × Source	0.220	0.385	0.711	0.033	<0.001

^a,b,c,d,e,A,B,C,D^ Means for treatments within a row not showing a common small letter superscript differ (*p* < 0.05), means for patterns within a row not showing a common capital letter superscript differ (*p* < 0.05), † different from the corresponding inorganic treatments (*p* < 0.05), the same as below. Control treatment (basic diet without add extra trace minerals, **CT**); NRC recommended level (**NRC-L**), inorganic trace minerals of NRC-L (**IN**), organic trace minerals of NRC-L pattern (**ON**), NY/T 33-2004 recommended level (**NY/T-L**), inorganic trace minerals of NY/T-L pattern (**IY**), organic trace minerals of NY/T-L pattern (**OY**); pattern 3, 50% NRC (1994) recommended level (**50% NRC-L**), inorganic trace minerals of 50% NRC-L pattern (**IHN**), organic trace minerals of 50% NRC-L pattern (**OHN**), the ratio of mineral elements in blood of laying hens was taken as the supplement proportion of trace elements, and Zn was supplemented depended on NRC recommended level (**TLB**), inorganic trace minerals of TLB pattern (**IB**), organic trace minerals of TLB pattern (**OB**).

**Table 8:** Effect of different patterns and sources of mineral elements on the spleen (mg/kg)^1^.

Items		Zn	Fe	Cu	Mn	Se
	CT	24.41	144.42	2.16	0.05	0.49 ^e^
NRC-L	IN	26.16	121.95	2.01	0.08	0.43 ^e^
	ON	26.89	143.67	2.14	0.06	0.53 ^de^
NY/T-L	IY	25.41	152.66	2.14	0.10	0.53 ^de^
	OY	23.17	129.15	2.01	0.07	0.65 ^bc^
50% NRC-L	IHN	25.38	141.62	2.04	0.06	0.52 ^de^
	OHN	24.35	144.09	2.04	0.08	0.60 ^cd^
TLB	IB	25.18	152.65	2.52	0.15	0.72 ^b^
	OB	23.61	172.98	2.11	0.06	0.94 ^a^
	SEM	0.66	3.84	0.05	0.01	0.03
Patterns	NRC-L	26.52	132.81	2.08	0.07	0.48 ^C^
	NY/T-L	24.29	140.91	2.08	0.08	0.59 ^B^
	50% NRC-L	24.86	142.86	2.04	0.07	0.56 ^BC^
	TLB	24.40	162.81	2.32	0.11	0.83 ^A^
Sources	Inorganic	25.53	54.85	2.18	0.10	0.55 ^†^
	Organic	24.51	60.77	2.07	0.07	0.68
*p*-value	CT-trace element supplemented	0.963	0.081	0.552	0.364	<0.001
	Pattern	0.753	0.063	0.265	0.510	<0.001
	Source	0.539	0.488	0.337	0.180	<0.001
	Pattern × Source	0.926	0.150	0.356	0.388	0.224

^a,b,c,d,e,A,B,C^ Means for treatments within a row not showing a common small letter superscript differ (*p* < 0.05), means for patterns within a row not showing a common capital letter superscript differ (*p* < 0.05), † different from the corresponding inorganic treatments (*p* < 0.05), the same as below. Control treatment (basic diet without add extra trace minerals, **CT**); NRC recommended level (**NRC-L**), inorganic trace minerals of NRC-L (**IN**), organic trace minerals of NRC-L pattern (**ON**), NY/T 33-2004 recommended level (**NY/T-L**), inorganic trace minerals of NY/T-L pattern (**IY**), organic trace minerals of NY/T-L pattern (**OY**); pattern 3, 50% NRC (1994) recommended level (**50% NRC-L**), inorganic trace minerals of 50% NRC-L pattern (**IHN**), organic trace minerals of 50% NRC-L pattern (**OHN**), the ratio of mineral elements in blood of laying hens was taken as the supplement proportion of trace elements, and Zn was supplemented depended on NRC recommended level (**TLB**), inorganic trace minerals of TLB pattern (**IB**), organic trace minerals of TLB pattern (**OB**).

**Table 9:** Effect of different patterns and sources of mineral elements on the pectoral muscles (mg/kg).

Items		Zn	Fe	Cu	Mn	Se
	CT	3.96	8.36	1.48	0.08	0.08 ^e^
NRC-L	IN	3.59	8.42	1.39	0.05	0.09 ^de^
	ON	3.25	6.87	1.45	0.07	0.12 ^cd^
NY/T-L	IY	3.90	8.08	1.38	0.06	0.11 ^cde^
	OY	3.37	7.01	1.33	0.05	0.20 ^b^
50% NRC-L	IHN	3.98	7.39	1.36	0.06	0.09 ^de^
	OHN	3.81	6.58	1.35	0.05	0.15 ^c^
TLB	IB	3.67	7.13	1.33	0.05	0.12 ^cd^
	OB	4.11	7.44	1.4	0.05	0.42 ^a^
	SEM	0.12	0.2	0.02	0.003	0.02
Patterns	NRC-L	3.42	7.55	1.42	0.06	0.10 ^C^
	NY/T-L	3.63	7.55	1.36	0.06	0.16 ^B^
	50% NRC-L	3.89	6.99	1.36	0.06	0.12 ^BC^
	TLB	3.89	7.29	1.37	0.05	0.27 ^A^
Sources	Inorganic	3.78	7.75	1.37	0.06	0.10 ^†^
	Organic	3.63	6.98	1.38	0.05	0.22
*p*-value	CT-trace element supplemented	0.738	0.343	0.668	0.156	<0.001
	Pattern	0.496	0.700	0.690	0.436	<0.001
	Source	0.552	0.072	0.752	0.792	<0.001
	Pattern × Source	0.558	0.443	0.745	0.168	<0.001

^a,b,c,d,e,A,B,C^ Means for treatments within a row not showing a common small letter superscript differ (*p* < 0.05), means for patterns within a row not showing a common capital letter superscript differ (*p* < 0.05), † different from the corresponding inorganic treatments (*p* < 0.05), the same as below. Control treatment (basic diet without add extra trace minerals, **CT**); NRC recommended level (**NRC-L**), inorganic trace minerals of NRC-L (**IN**), organic trace minerals of NRC-L pattern (**ON**), NY/T 33-2004 recommended level (**NY/T-L**), inorganic trace minerals of NY/T-L pattern (**IY**), organic trace minerals of NY/T-L pattern (**OY**); pattern 3, 50% NRC (1994) recommended level (**50% NRC-L**), inorganic trace minerals of 50% NRC-L pattern (**IHN**), organic trace minerals of 50% NRC-L pattern (**OHN**), the ratio of mineral elements in blood of laying hens was taken as the supplement proportion of trace elements, and Zn was supplemented depended on NRC recommended level (**TLB**), inorganic trace minerals of TLB pattern (**IB**), organic trace minerals of TLB pattern (**OB**).

**Table 10:** Effect of different patterns and sources of mineral elements on the tibia (mg/kg) ^1^.

Items		Zn	Fe	Cu	Mn	Se
	CT	108.01	78.42	1.85	2.43 ^ab^	0.11 ^c^
NRC-L	IN	124.02	69.84	1.82	3.62 ^ab^	0.11 ^c^
	ON	122.51	81.94	1.96	3.27 ^ab^	0.14 ^c^
NY/T-L	IY	128.29	77.79	1.82	4.07 ^ab^	0.14 ^c^
	OY	120.86	64.58	2.06	4.37 ^a^	0.20 ^b^
50% NRC-L	IHN	131.4	79.73	1.95	3.30 ^ab^	0.13 ^c^
	OHN	115.66	72.75	1.87	3.84 ^ab^	0.14 ^c^
TLB	IB	122.97	88.88	1.84	2.93 ^ab^	0.23 ^b^
	OB	116.21	72.03	1.76	2.26 ^b^	0.40 ^a^
	SEM	1.91	2.02	0.05	0.16	0.02
Patterns	NRC-L	123.26	75.90	1.89	3.57 ^AB^	0.12 ^C^
	NY/T-L	124.57	71.19	1.94	4.22 ^A^	0.17 ^B^
	50% NRC-L	123.53	76.24	1.91	3.57 ^AB^	0.13 ^BC^
	TLB	119.59	80.46	1.80	2.60 ^B^	0.31 ^A^
Sources	Inorganic	126.67	70.06	1.86	3.48	0.15 ^†^
	Organic	118.81	72.83	1.91	3.43	0.22
*p*-value	CT-trace element supplemented	0.156	0.277	0.943	0.025	<0.001
	Pattern	0.807	0.448	0.838	0.011	<0.001
	Source	0.052	0.144	0.244	0.882	<0.001
	Pattern × Source	0.665	0.100	0.496	0.500	<0.001

^a,b,c,A,B,C^ Means for treatments within a row not showing a common small letter superscript differ (*p* < 0.05), means for patterns within a row not showing a common capital letter superscript differ (*p* < 0.05), † different from the corresponding inorganic treatments (*p* < 0.05), the same as below. Control treatment (basic diet without add extra trace minerals, **CT**); NRC recommended level (**NRC-L**), inorganic trace minerals of NRC-L (**IN**), organic trace minerals of NRC-L pattern (**ON**), NY/T 33-2004 recommended level (**NY/T-L**), inorganic trace minerals of NY/T-L pattern (**IY**), organic trace minerals of NY/T-L pattern (**OY**); pattern 3, 50% NRC (1994) recommended level (**50% NRC-L**), inorganic trace minerals of 50% NRC-L pattern (**IHN**), organic trace minerals of 50% NRC-L pattern (**OHN**), the ratio of mineral elements in blood of laying hens was taken as the supplement proportion of trace elements, and Zn was supplemented depended on NRC recommended level (**TLB**), inorganic trace minerals of TLB pattern (**IB**), organic trace minerals of TLB pattern (**OB**).

**Table 11:** Effect of different patterns and sources of mineral elements on fecal mineral concentration (mg/kg).

Items		Zn	Fe	Cu	Mn	Se
	CT	56.20 ^d^	996.36 ^b^	16.03 ^d^	60.99 ^d^	0.40 ^d^
NRC-L	IN	111.16 ^bc^	918.92 ^b^	22.46 ^cd^	94.17 ^b^	0.19 ^e^
	ON	136.57 ^b^	931.43 ^b^	24.16 ^bc^	104.42 ^b^	0.16 ^e^
NY/T-L	IY	221.05 ^a^	1011.9 ^b^	33.27 ^a^	186.76 ^a^	0.63 ^c^
	OY	231.91 ^a^	1045.78 ^ab^	35.52 ^a^	180.61 ^a^	0.55 ^c^
50% NRC-L	IHN	121.60 ^bc^	893.42 ^b^	21.51 ^cd^	93.09 ^bc^	0.17 ^e^
	OHN	95.64 ^c^	1024.76 ^b^	21.09 ^cd^	82.36 ^bcd^	0.11 ^e^
TLB	IB	117.33 ^bc^	1219.53 ^a^	30.51 ^ab^	64.14 ^cd^	0.81 ^b^
	OB	125.60 ^b^	1065.96 ^ab^	36.05 ^a^	60.62 ^d^	1.55 ^a^
	SEM	7.73	17.84	1.05	6.64	0.09
Patterns	NRC-L	123.86 ^B^	925.18 ^B^	23.31 ^B^	99.29 ^AB^	0.18 ^C^
	NY/T-L	226.48 ^A^	1028.86 ^AB^	34.40 ^A^	183.69 ^A^	0.59 ^B^
	50% NRC-L	108.62 ^B^	959.09 ^B^	21.30 ^B^	87.73 ^AB^	0.14 ^C^
	TLB	121.46 ^B^	1142.75 ^A^	33.28 ^A^	62.38 ^B^	1.18 ^A^
Sources	Inorganic	142.78	1010.96	26.94 ^†^	109.54	0.45 ^†^
	Organic	147.43	1016.98	29.21	107.00	0.59
*p*-value	CT-trace element supplemented	<0.001	<0.001	<0.001	<0.001	<0.001
	Pattern	<0.001	<0.001	<0.001	<0.001	<0.001
	Source	0.342	0.837	0.043	0.566	<0.001
	Pattern × Source	0.004	0.013	0.303	0.365	<0.001

^a,b,c,d,e,A,B,C^ Means for treatments within a row not showing a common small letter superscript differ (*p* < 0.05), means for patterns within a row not showing a common capital letter superscript differ (*p* < 0.05), † different from the corresponding inorganic treatments (*p* < 0.05), the same as below. Control treatment (basic diet without add extra trace minerals, **CT**); NRC recommended level (**NRC-L**), inorganic trace minerals of NRC-L (**IN**), organic trace minerals of NRC-L pattern (**ON**), NY/T 33-2004 recommended level (**NY/T-L**), inorganic trace minerals of NY/T-L pattern (**IY**), organic trace minerals of NY/T-L pattern (**OY**); pattern 3, 50% NRC (1994) recommended level (**50% NRC-L**), inorganic trace minerals of 50% NRC-L pattern (**IHN**), organic trace minerals of 50% NRC-L pattern (**OHN**), the ratio of mineral elements in blood of laying hens was taken as the supplement proportion of trace elements, and Zn was supplemented depended on NRC recommended level (**TLB**), inorganic trace minerals of TLB pattern (**IB**), organic trace minerals of TLB pattern (**OB**).

## Data Availability

The data presented in this study are available upon request from the corresponding author.
